# Physicochemical, Antibacterial Properties, and Compatibility of ZnO-NP/Chitosan/β-Glycerophosphate Composite Hydrogels

**DOI:** 10.4014/jmb.2111.11024

**Published:** 2022-01-07

**Authors:** Pingping Huang, Wen Su, Rui Han, Hao Lin, Jing Yang, Libin Xu, Lei Ma

**Affiliations:** 1 The Affiliated Hospital of Qingdao University, Qingdao 266003, P.R. China; 2 School of Stomatology of Qingdao University, Qingdao 266003, P.R. China

**Keywords:** Hydrogel, chitosan, ZnO-NPs, antibacterial

## Abstract

In this study we aimed to develop novel ZnO-NP/chitosan/β-glycerophosphate (ZnO-NP/CS/β-GP) antibacterial hydrogels for biomedical applications. According to the mass fraction ratio of ZnO-NPs to chitosan, mixtures of 1, 3, and 5% ZnO-NPs/CS/β-GP were prepared. Using the test-tube inversion method, scanning electron microscopy and Fourier-transform infrared spectroscopy, the influence of ZnO-NPs on gelation time, chemical composition, and cross-sectional microstructures were evaluated. Adding ZnO-NPs significantly improved the hydrogel's antibacterial activity as determined by bacteriostatic zone and colony counting. The hydrogel's bacteriostatic mechanism was investigated using live/dead fluorescent staining and scanning electron microscopy. In addition, crystal violet staining and MTT assay demonstrated that ZnO-NPs/CS/β-GP exhibited good antibacterial activity in inhibiting the formation of biofilms and eradicating existing biofilms. CCK-8 and live/dead cell staining methods revealed that the cell viability of gingival fibroblasts (L929) cocultured with hydrogel in each group was above 90% after 24, 48, and 72 h. These results suggest that ZnO-NPs improve the temperature sensitivity and bacteriostatic performance of chitosan/β-glycerophosphate (CS/β-GP), which could be injected into the periodontal pocket in solution form and quickly transformed into hydrogel adhesion on the gingiva, allowing for a straightforward and convenient procedure. In conclusion, ZnO-NP/CS/β-GP thermosensitive hydrogels could be expected to be utilized as adjuvant drugs for clinical prevention and treatment of peri-implant inflammation.

## Introduction

Peri-implants are a new restoration method in dentistry, which can replace missing teeth and restore masticatory, occlusal, and aesthetic functions [1]. In a long-term clinical study of 10,871 dental implants followed for 22 years, the incidence of peri-implant mucositis and peri-implant inflammation was 11.9% and 7.1% at 8-10 years, respectively [2]. Peri-implantitis is an inflammatory disease that affects soft and hard tissues around teeth and functioning implants [3] and is mainly manifested by alveolar bone resorption, even implant loosening, and loss [4]. *Porphyromonas gingivalis* (*P. gingivalis*) is typically considered the “late colonizer” of subgingival biofilms and is associated with periodontal diseases such as peri-implantitis [5]. *P. gingivalis* secretes large amounts of virulence factors and gingipains, resulting in a high concentration of inflammatory factors such as IL-1β and IL-6, causing periodontal tissue destruction [6].

The efficacy of plaque control largely determines the prognosis of peri-implantitis. Clinically, basic periodontal treatment, periodontal surgery, and drug treatment (mostly antibiotics) are often used to improve pathogenic bacteria removal as well as periodontitis and peri-implantitis treatment [7]. However, traditional antibiotics are restricted by the induction of antibiotic resistance and their low efficiency to kill bacteria in biofilms, which imposes certain limitations on clinical applications [8, 9]. Identifying a biological agent that does not produce bacterial resistance and inhibits biofilm formation is important.

Chitosan (CS), derived from chitin, is a naturally occurring polysaccharide with outstanding biocompatibility, biodegradability, and antibacterial activity [10]. However, to some extent, CS applications are limited due to the high viscosity and low solubility of CS [11]. Many chemical groups on the chain of CS molecules can be involved in the reaction and can also be further modified as required [12]. In recent years, composite hydrogels based on CS and nanomaterials have demonstrated significant potential and utility in food, medical and other fields due to their advantages of improving thermal stability, antibacterial activity, and the mechanical tensile strength of CS [13, 14]. Hoomaan *et al*. increased the biocompatibility of GO nanoparticles by adding CS and disclosed that the synthesized nano-hybrids exhibit higher bactericidal and bio-safety performance [15]. Wang *et al*. demonstrated for the first time that CS@Fe_3_O_4_ nanoparticles had an inhibitory effect on *Acinetobacter baumannii* [16].

As new inorganic nanomaterials, ZnO-NPs have garnered considerable attention in the field of stomatology due to their excellent antibacterial performance, long-lasting antibacterial effect, and good biocompatibility [17] . Existing in vitro antibacterial tests have revealed that ZnO-NPs exhibited good inhibitory activity against common oral pathogens, such as *Streptococcus mutans*, *P. gingivalis*, and *Actinobacillus actinomycetemcomitans* [18]. In previous studies, ZnO-NPs were added into a composite resin, binder, and denture base resin and attached to the implant's surface with the results indicating that the number of bacteria and biofilm formation could be reduced to a certain extent [19]. In this study, we added ZnO-NPs into CS to synthesize antimicrobial hydrogels, and we investigated the effects of varying ZnO-NP content on gelation time, morphology, antibacterial activity, and compatibility. The bactericidal mechanism of hydrogels on *P. gingivalis* was studied using live/dead fluorescent staining and scanning electron microscopy. Our synthetic hydrogels provide a promising approach for clinical applications in treating peri-implant inflammation.

## Materials and Methods

### Chemicals

CS and β-glycerophosphate were obtained from Solarbio (China). ZnO-NPs (20-30 nm) and acetic acid were purchased from Maclean Biochemical (China). Calcein-AM/PI solution, phosphate-buffered saline (PBS), and a Live/Dead Bacterial staining kit were supplied by Meilunbio (China). Cell Counting Kit-8 (CCK8) was provided by MedChemExpress (USA). MTT reagent, crystal violet stain solution, and brain–heart infusion broth (BHI) were purchased from Hopebio (China). Penicillin, streptomycin, fetal bovine serum, and minimum essential medium (MEM) were obtained from Biological Industries (Israel).

### Preparation of Thermosensitive Hydrogel

In this work, ZnO-NPs with a particle size of 20-30 nm were purchased and tested. At room temperature, 200 mg CS was weighed and dissolved in 10 ml 0.1 mol/l acetic acid solution. ZnO-NPs at weights of 0, 2, 6, and 10 mg were added, respectively, followed by ultrasonication for 1 min. About 4.24 g β-GP was weighed and dissolved in 5 ml of deionized water. CS, 1, 3, and 5% ZnO-NPs/CS, and 56% (w/v) β-GP were mixed under continuous magnetic agitation at a ratio of 7:3 under an ice-water bath.

### Characterization of Hydrogel

The gelation time of hydrogel was investigated using the test-tube inversion method [20]. One milliliter of liquid hydrogel was charged into a test tube and placed at a 37°C constant temperature water bath. The tube was turned upside down every 30 s to observe flow performance. If no flow occurs for 15 s, the solution has been hydrogelated, and the time required to change the hydrogel from liquid to solid at this temperature is called the gelation time. The liquid pH of hydrogel was measured using a pH meter.

Transmission electron microscopy (TEM) was performed using HITACHI (H -7650, Japan) to determine the exact size of the ZnO-NPs. The hydrogel was prepared and observed using scanning electron microscopy (SEM)(Germany), and SEM images were obtained for analysis. Fourier-transform infrared spectroscopy (FTIR)(Thermo Nicolet Corp, USA) was primarily employed to identify the hydrogel's chemical structure.

### Bacterial Strain and Culture Conditions

*P. gingivalis* (ATCC 33277) was anaerobically cultivated with BHI medium (80% N_2_, 10% H_2_, and 10% CO_2_) at 37°C for 48 h. Based on the standard curve of OD600 nm, the concentration of bacterial liquid in the reserve solution was adjusted to 1.0 × 10^8^ CFU/ml.

### Bacteriostatic Ring Experiment

Using a coating stick, 100 μl (1.0 × 10^8^ CFU/ml) of the bacterial solution was uniformly spread over solidified nutrient agar. The drug-sensitive papers with a diameter of 6 mm were immersed in the hydrogel for 5 min before being affixed to the surface of the bacteria-containing culture medium using sterile forceps and incubated at 37°C for 48 h. The antagonistic activities of samples were quantified by measuring the inhibition zone surrounding sensitive papers [21]. The experiments were conducted in triplicate, and the average was taken as standard error.

### Mini-Dilution and Antibacterial Rate

The hydrogel was first formed in 96-well plates, followed by the addition of 100 μl of *P. gingivalis* suspension (1.0×108 CFU/ml). The 96-well plates containing hydrogel and bacteria were shaken at 37°C for 24 h. Anhydrous hydrogel-treated bacterial solution was used as a control, and each group contained three sub-wells. After 24 h, the bacterial solution was diluted according to varying concentration gradients, and AGAR plates were evenly coated. After two days of culture, colonies were counted, and the antibacterial rate was calculated as follows: antibacterial rate = (Nc-Ns)/Nc*100% (Nc and Ns were the numbers of colonies on AGAR plates in control and hydrogel groups, respectively).

### Morphology Investigation of Bacteria

After contact with different hydrogel groups, the bacterial morphology was investigated using SEM. Briefly, after 4 h contact with hydrogel, *P. gingivalis* was dropped onto silicon wafers, fixed with 2% glutaraldehyde, and gradually dehydrated using a series of ethanol solutions. Finally, silicon wafers with bacteria were dried and imaged using SEM after platinum sputter coating.

### Live/Dead Fluorescent Staining

The viability of bacteria following contact with hydrogels was investigated using a live/dead fluorescent staining assay. Briefly, before and after hydrogel treatment, bacteria were mixed with dye solution comprising SYTO 9 and propidium iodide for 15 min at room temperature and then imaged using confocal microscopy. According to the manufacturer’s instructions, live bacterial cells were stained using SYTO 9 dye (green color), while dead bacterial cells were labeled using propidium iodide dye (red color) due to damaged cell wall and membrane. Three images were randomly selected from each specimen of each group with three bacterial species.

### Biofilm Formation Evaluation by Crystal Violet Staining

CS/β-GP and ZnO-NPs/CS/β-GP were first formed in 24-well plates, followed by the addition of *P. gingivalis* suspensions (1.0 × 10^8^ CFU/ml). After two days of incubation, the plates were gently washed three times using PBS, followed by the addition of 4% paraformaldehyde and fixation for 15 min. Following that, 500 μl crystal violet was added for staining for 20 min, and 500 μl ethanol was added for 20 min after washing with PBS. The absorbance of the corresponding crystal violet solution at 590 nm (A590 nm) was measured using a microplate reader.

### Biofilm Eradication by MTT Assay

MTT assay was used to determine the metabolic activity of biofilms [22]. *P. gingivalis* suspensions (1.0 × 10^8^ CFU/ml) were added to a 24-well plate and allowed to grow for two days, forming an integrated biofilm. The supernatant was aspirated from the 24-well plate and gently rinsed with PBS to remove suspended bacteria. The sterilized hydrogel in each group was cut into small pieces, placed on the surface of the mature biofilm, and incubated for 4 h. After 4 h, the hydrogel in the 24-well plate was sucked off, and the biofilm surface was washed with PBS. Each group of biofilms was treated with MTT dye at 37°C in 5% CO_2_ for 4 h. Following that, formazan crystals could be observed at the bottom of wells and were treated with equivalent dimethyl sulfoxide (DMSO). The absorbance values were measured at OD490 nm using a microplate reader.

### Cytocompatibility Assessment

Mouse fibroblasts (L929) used to evaluate the cytocompatibility of thermosensitive hydrogel were provided by Qingdao University laboratory. According to ISO 10993-5, the material weight ratio to extraction medium volume is 0.2 g/ml. About 10% serum-containing MEM medium was added to the hydrogel and extracted at 37°C for 24 h, and then extraction media was collected using a 0.2 μm filter. The cell suspension with a density of (5-10) × 10^4^ cells/ml was added to the 24-well plate at 0.5 ml per well and incubated overnight. After discarding the culture medium, the extraction media was added for further culture for 24, 48, and 72 h. The cells cultured in MEM medium containing 10% serum were used as a blank control. Following incubation, the cells were washed with PBS to eliminate non-adherent cells and media. CCK-8 reagent was added to each well and incubated at 37°C for 1 h. The cytocompatibility of L929 cells was determined at 450 nm according to the manufacturer's instructions.

### Living/Dead Cell Staining

Following the above steps, mouse fibroblasts (L929) were cocultured with the extraction media for 24, 48, and 72 h and then rinsed with PBS. Calcein-AM/PI solution was diluted and added to a 24-well plate to protect from light for 0.5 h incubation. Calcein-AM can penetrate the living cell membrane and generate strong green fluorescence through the cell's esterase action. On the other hand, PI cannot penetrate the living cell membrane, but when the cell membrane is damaged, PI enters the cell and combines with a nucleic acid to produce bright red fluorescence. The morphology of stained cells and living/dead cells were observed under a fluorescence microscope equipped with excitation light at 490 nm and 545 nm wavelengths.

### Statistical Analysis

GraphPad InStat statistical software was employed for statistical analysis, and a single-factor square was utilized to analyze experimental data. *p* < 0.05 was considered statistically significant.

## Results

### Temperature-Sensitive Characteristics

**Fig. 1:**
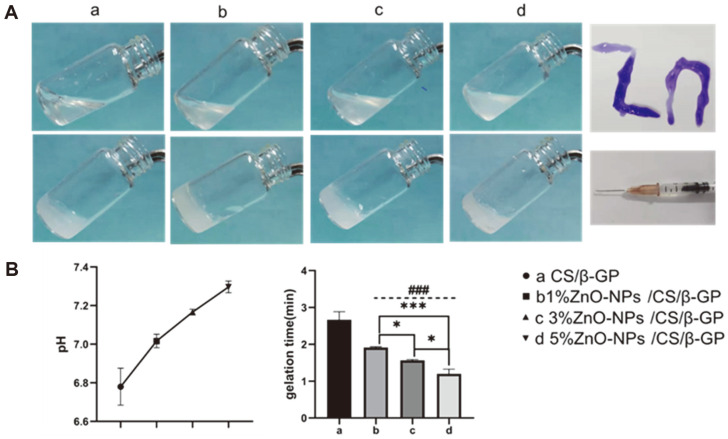
Temperature-sensitive characteristics of hydrogels. (**A**) Temperature sensitivity and injectable property of hydrogels. (**B**) pH of hydrogels. (**C**) Gelation time of hydrogels. Error bars represent ± standard error of the mean. ^###^*p* < 0.001 represents ZnO-NPs/CS/β-GP versus CS/β-GP, whereas *p < 0.05 and ***p < 0.001 represent comparison between ZnO-NP/ CS/β-GP groups.

The transparent liquid hydrogel prepared at room temperature can gel rapidly in a constant-temperature water bath at 37°C and exhibits good temperature sensitivity and injectability (Fig. 1). The average gelation time of CS/β-GP was 2.66 min, and the average gelation times of 1, 3, and 5% ZnO-NPs/CS/β-GP were 1.91, 1.56, and 1.19 min, respectively (*p* < 0.05). The data indicated that the hydrogel-formation time was shorter when ZnO-NP content was higher at 37°C. Compared with CS/β-GP, the pH of hydrogels containing ZnO-NPs increases but remains within the neutral range.

### SEM Images

**Fig. 2:**
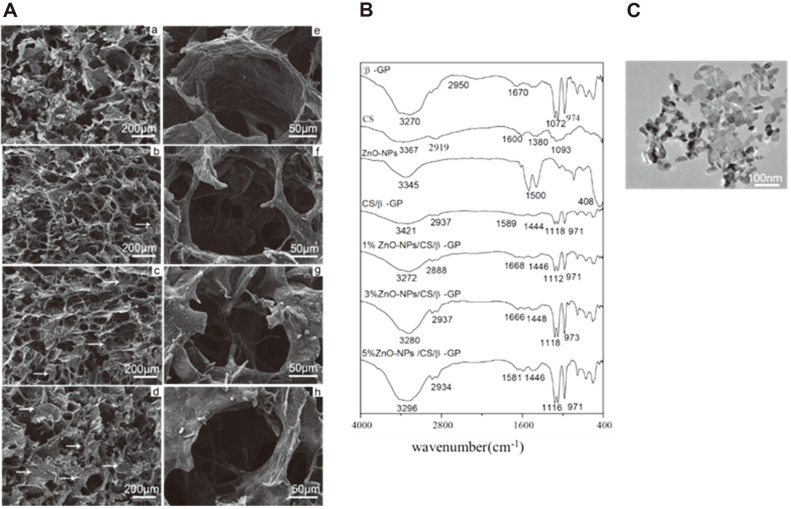
SEM images and FTIR analysis of hydrogels. (**A**) SEM images. (**B**) FTIR spectrogram. (**C**) TEM image confirmed that ZnO-NPs are formed as particles approximately 20-30 nm in diameter.

SEM was used to determine the cross-section of hydrogels. As depicted in Fig. 2A, SEM observation reveals that all hydrogels possess three-dimensional porous network structures with pore sizes ranging between 100 and 250 μm. In the ZnO-NPs/CS/β-GP group, micro-nano particles are evenly embedded in the hydrogel's pore wall (Fig. 2A, indicated by the arrow). ZnO-NPs have good biocompatibility, and adding ZnO-NPs to CS/β-GP did not cause significant changes in the hydrogel's original pore size and shape.

### FTIR Analysis

The changes in absorption peak and stretching vibration of CS/β-GP and ZnO-NPs/CS/β-GP were analyzed by FTIR (Fig. 2B). In CS/β-GP infrared spectrum, a wide absorption peak at 3,421 cm^-1^ was attributed to the combined vibration of O-H and N-H. The absorption peaks at 2,937, 1,589, and 1,118 cm^-1^ correspond to tensile vibration of C-H, bending vibration of N-H, and tensile vibration of C6-OH, respectively. After adding ZnO-NPs, the infrared spectra remained relatively unchanged, but the expansion joint at 3,421 cm^-1^ strengthened and shifted to a low wavenumber. The intermolecular hydrogen bonding force of composite hydrogels was enhanced, contributing to the formation of complex hydrogels with dense and high condensation strength [23]. The results showed that composite hydrogels did not produce a significant new absorption peak, indicating that ZnO-NPs had no significant influence on the structure of composite hydrogels.

### Determination of Antimicrobial Activity

**Fig. 3:**
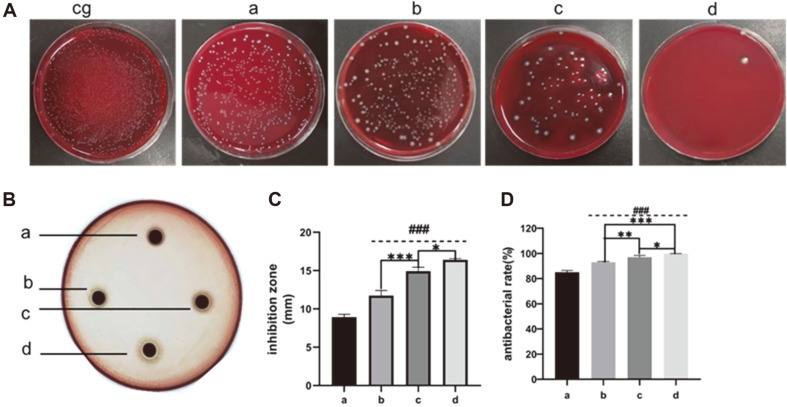
The antimicrobial effects of hydrogels. (**A**) Representative photographs of bacteria after direct contact with hydrogels for 24 h. (**B**) Representative photographs of inhibition zones of hydrogels. (**C**) Bacteriostatic zones of hydrogels. (**D**) Antibacterial effects of hydrogels. Error bars represent ± standard error of the mean. ^###^*p* < 0.001 represents ZnO-NPs/CS/β-GP versus CS/β-GP, whereas *p < 0.05, **p < 0.01, and ***p < 0.001 represent comparison between ZnO-NPs/CS/β-GP groups.

ZnO-NP/CS/β-GP composite hydrogels exhibit an antibacterial effect on *P. gingivalis* (Fig. 3). We evaluated the antibacterial activity of hydrogels in detail using two different approaches; one of them was the conventional inhibition zone method [24]. Fig. 3BC shows bacteriostatic zones for different hydrogels. It can be seen that the diameters of antibacterial rings of 1, 3, and 5% ZnO-NPs/CS/β-GP were 16.4, 14.9, and 11.7 mm, respectively, which were significantly larger than CS/β-GP's bacteriostatic zone (8.9 mm). This indicates that adding ZnO-NPs significantly improved the antibacterial activity of CS/β-GP, and the bacteriostatic zone increased with increasing ZnO-NP content (*p* < 0.05).

In addition, to test the hydrogel's potential applicability, we employed another test method based on the direct contact between hydrogel and bacteria [25]. Based on the hydrogel's antibacterial effect on free bacteria (Fig. 3AD), the number of colonies on the ZnO-NP/CS/β-GP agar plate was significantly less than that on CS/β-GP, and almost no *P. gingivalis* growth was observed in 5% ZnO-NPs/CS/β-GP petri dish. The antibacterial rate of 5% ZnO-NPs/CS/β-GP against *P. gingivalis* was 99.7%, indicating its excellent antibacterial effect. Additionally, CS/β-GP has antibacterial potential, although this activity is lower than composite hydrogels containing ZnO-NPs. Overall, ZnO-NPs/CS/β-GP is superior to CS/β-GP in antimicrobial activity and shows a bigger bacteriostatic zone and higher antibacterial rate.

### Antimicrobial Mechanism of ZnO-NP/CS/β-GP Hydrogel

**Fig. 4:**
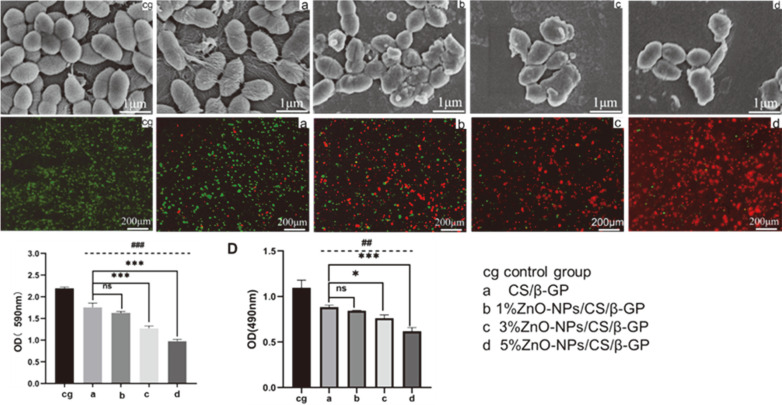
Antimicrobial mechanism of hydrogel and effect of hydrogel on biofilm. (**A**) SEM images for bacterial morphology incubated with different hydrogels. (**B**) Overlapping fluorescent images of live/dead staining incubated with different hydrogels. (**C**) Results for the effects of crystal violet treatment with different hydrogels on biofilm formation of *P. gingivalis*. (**D**) Results of metabolic activity with different hydrogels on integrated biofilm eradication. Error bars represent ± standard error of the mean. ^###^*p* < 0.001 represents ZnO-NPs/CS/β-GP versus CS/β-GP, whereas *p < 0.05 and ***p < 0.001 represent comparison between ZnO-NPs/CS/β-GP groups.

The specific mechanism underlying the antibacterial activity of hydrogels was subsequently investigated using live/dead fluorescent staining and bacterial morphology with SEM (Fig. 4AB). The bacteria in the control group had typical bacteria morphology, with sharp edges and a smooth shape. The morphology of bacteria treated with CS/β-GP remained in the shape of ball rods, and damage to the bacterial edges could be observed. The bacteria treated with ZnO-NP/CS/β-GP hydrogels were deformed, and their surface was obviously collapsed, exhibiting rough and serrated edges. The damage caused by ZnO-NPs/CS/β-GP to the morphology and integrity of *P. gingivalis* was more significantly apparent than that of CS/β-GP. This phenomenon indicates that the negative charge on the bacterial surface attracts ZnO-NPs by electrostatic interactions, which destroy the morphology of bacteria and the membrane integrity and ultimately lead to bacterial death.

Live/dead fluorescent staining was employed to determine bacterial viability using green fluorescent dye SYT09 and red fluorescent dye PI (propyl iodide). Bacteria with intact cell membranes exhibited green fluorescence, while bacteria with damaged cell membranes revealed red fluorescence. As depicted in Fig. 4B, only green fluorescence was emitted in the control group, and a small amount of red fluorescence was emitted in the CS/β-GP group. The red fluorescence of the ZnO-NP/CS/β-GP hydrogel was significantly higher than that of CS/β-GP hydrogel. Almost all bacteria in 5% ZnO-NP/CS/β-GP hydrogel were labeled red, indicating that they were all dead. The results were consistent with those of bacterial morphology experiments and further confirmed the antibacterial mechanism of hydrogels in destroying bacterial cell membranes.

### Effect of Hydrogel on Biofilm

Fig. 4C compares the amount of biofilm formation in each group using crystal violet staining. All hydrogels showed a significant effect against *P. gingivalis* biofilm formation (*p* < 0.001). The total A590 nm of 1% ZnO-NP/CS/GP biofilm was 1.63, revealing no significant difference compared with the total A590 nm of CS/GP biofilm (*p* > 0.05). The total amount of biofilm formation in 3% and 5% ZnO-NP/CS/β-GP groups was 1.27 and 0.97, respectively, significantly lower than that in CS/GP group (*p* < 0.001). This study revealed a significant difference in biofilm inhibition of 3% and 5% ZnO-NP synthetic hydrogels compared to CS/GP, consistent with other studies in which ZnO-CS nanoparticles were incorporated into dentine bonding agents, exhibiting bacteriostatic effects [26].

Fig. 4D illustrates a study on metabolic activity in each group of integrated biofilms, which was employed to compare biofilm eradication by hydrogels. There was no significant difference in metabolic activity between biofilms comprising 1% ZnO-NPs/CS/GP and CS/GP (*p* > 0.05). The metabolic activity of integrated biofilms treated with 3% and 5% ZnO-NPs/CS/β-GP was significantly lower than that of CS/GP (*p* < 0.05). Moreover, 5%ZnO-NPs/CS/β-GP can reduce biofilm activity by nearly half. The results indicated that 3% and 5% ZnO-NPs/CS/β-GP had a good effect on the eradication of *P. gingivalis* biofilm. It was demonstrated that adding a small amount of ZnO-NPs (1%) did not improve the effect of the original hydrogel on inhibiting biofilm formation and eradication.

### Cell Compatibility

**Fig. 5:**
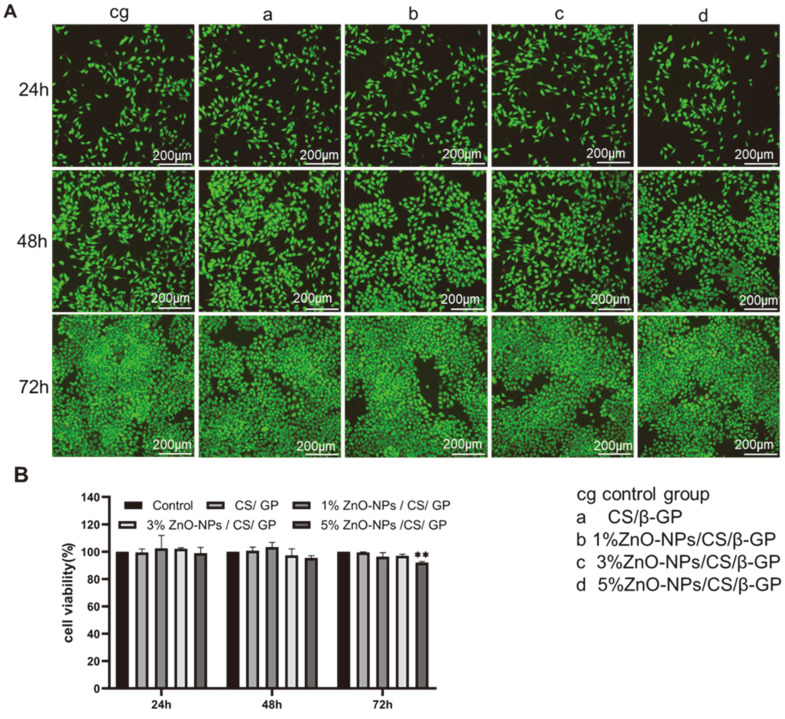
Cell compatibility of hydrogel. (**A**) Viability of L929 cells after incubation with hydrogel extracts for 24, 48, and 72 h. (**B**) Fluorescence microscopy images for L929 cells cocultured with different hydrogel extracts treated with live/dead cell assay at 24, 48, and 72 h. Error bars represent ± standard error of the mean. **p < 0.01 represents 5% ZnO-NPs/CS/β-GP versus CS/β-GP.

L929 was cultured for 24, 48, and 72 h in hydrogel extracts of each group, and the cell viability was tested using the CCK-8 method (Fig. 5A). When cells were cocultured with the hydrogel for 24 and 48 h, no significant difference existed in the cell viability of L929 in hydrogel extracts of each group compared with the control group. After 72 h of culture, the cell survival rate of L929 in 5% ZnO-NP/CS/β-GP hydrogel extract decreased, different from the control group (*p* < 0.01), but the cell viability remained greater than 90%. The hydrogels of each group revealed no apparent cytotoxicity toward L929 cells.

The cells were cocultured with hydrogel extracts for 24, 48, and 72 h, and the cell activity and growth were observed under an inverted fluorescence microscope (Fig. 5B). We observed that the cells of all experimental groups had clear contours, were fusiform, and had a good spreading shape. Most cells cultured with hydrogel extracts exhibited green fluorescence, and few showed red fluorescence. A little red fluorescence could be observed in the cell group cocultured with 5% ZnO-NP/CS/β-GP extract for 72 h, indicating that 5% ZnO-NPs/CS/β-GP had low cytotoxicity, which further confirmed its good cytocompatibility.

## Discussion

CS/β-GP thermosensitive hydrogel exhibits good fluidity at or below room temperature and could undergo rapid hydrogelation transformation at 37°C while maintaining stable thermosensitive performance [27]. When ZnO-NPs are added to a CS/β-GP hydrogel system, the hydrogelation time at body temperature (37°C) gradually decreases as ZnO-NP content increases, and the hydrogelation time of 5% ZnO-NPs/CS/β-GP at 37°C is only 71 s. Studies have proved that ZnO-NPs can bind CS molecules via metal ionic bonds and induce crosslinking, thus shortening the hydrogelation time and improving the hydrogel's mechanical properties [28]. In addition, SEM and FTIR demonstrated that ZnO-NPs could blend into CS hydrogel without affecting its structure or surface properties.

The composite hydrogels synthesized in this study had good cell compatibility, and the fibroblast showed an upward trend in proliferation, with normal cell morphology and without evidence of cytotoxicity. However, 5%ZnO-NPs/CS/β-GP exhibited minimal cytotoxicity after 72 h coculture with cells. Chen *et al*. demonstrated that a high concentration of ZnO-NPs caused toxicity to human gingival fibroblast cells and inhibited cell proliferation by regulating MDM2 and p53 expression [29]. Jayasuriya *et al*. observed significant cytotoxicity in CS films with ZnO-NP content higher than 5% (w/w) [30]. On the contrary, Jingyu *et al*. confirmed that ZnO-NPs increased the expression of OCCM-30 osteogenesis-related factors Bsp and Runx2 [31]. Careta *et al*. found that a ZnO-NP coating on the surface of Ti alloy is non-cytotoxic and could promote the expression of early differentiation genes of osteoblasts without external stimulation [32]. Therefore, we should verify the cytotoxicity of synthesized hydrogels in further investigations and select a compound hydrogel with better antibacterial performance and no cytotoxicity.

In this work, 20-30 nm ZnO-NPs were added to CS, which greatly improved the antibacterial activity of CS hydrogel and inhibited biofilm formation. Existing literature has revealed that ZnO-NPs with a size range of 10-50 nm exhibit superior antibacterial properties, particularly with small-sized particles [33]. SEM revealed that ZnO-NP/CS/β-GP-treated bacteria collapsed and exhibited an irregular shape, and the surface structure was completely destroyed because ZnO-NPs can be electrostatically attracted to negative charges on the bacterial membrane surface, which increases the contact area between hydrogel and bacteria, resulting in cell membrane damage and leakage of contents. CS alone has an antibacterial effect on *P. gingivalis*, possibly due to ionic interactions between some residual positively charged amino groups in CS and bacteria surface molecules leading to bacterial death [34]. Compared with CS/β-GP, ZnO-NP/CS/β-GP composite hydrogel exhibits higher bacteriostatic efficiency, as ZnO-NPs destroy bacterial cell walls more effectively.

During our studies, *P. gingivalis* was chosen as a model microorganism for antibacterial and biofilm formation studies. *P. gingivalis* was previously demonstrated to colonize titanium surfaces in multiple interconnected layers, forming a biofilm [35]. A 3% and 5% ZnO-NPs/CS/β-GP showed good antibacterial activity in inhibiting biofilm formation and eradicating existing biofilms. Bacterial biofilm is a membranous structure formed by bacteria embedded in a matrix of polysaccharides, proteins, and minerals. The pathogenicity and drug resistance of pathogenic bacteria in biofilms are 500 times higher than those of free bacteria, and the formation of pathogenic plaque biofilm is the initiating factor of peri-implantitis and periodontitis [25]. Minocycline hydrochloride is often used clinically to treat periodontitis and peri-implantitis, but it is prone to drug resistance, can only kill bacteria, and does not remove plaque biofilm, posing limitations in clinical applications [34]. The antibacterial hydrogel synthesized in this study can absorb tissue exudate, inhibit biofilm formation, and effectively remove biofilm, making it a strong candidate for bacterial removal and wound-healing intervention. This investigation illuminated the imperative aspects of the antimicrobial properties of ZnO-NPs/CS/β-GP in peri-implantitis for the first time and validated its potential as a hydrogel with good temperature sensitivity and injectable properties in clinical settings.
